# Improved detection of metastatic lymph nodes in oesophageal squamous cell carcinoma by combined interpretation of fluorine-18-fluorodeoxyglucose positron-emission tomography/computed tomography

**DOI:** 10.1186/s40644-019-0225-5

**Published:** 2019-06-21

**Authors:** Ji Young Lee, Young Hwan Kim, Yong-Jin Park, Soo Bin Park, Hyun Woo Chung, Jae Il Zo, Young Mog Shim, Kyung Soo Lee, Joon Young Choi

**Affiliations:** 1 Department of Nuclear Medicine, Jeju National University Hospital, Jeju National University School of Medicine, Jeju, Republic of Korea; 2 Department of Nuclear Medicine, Kangbuk Samsung Hospital, Sungkyunkwan University School of Medicine, Seoul, Republic of Korea; 3 Department of Nuclear Medicine, Samsung Medical Center, Sungkyunkwan University School of Medicine, 81 Irwon-ro, Gangnam-gu, Seoul, 06351 Republic of Korea; 4 Department of Radiology, Soonchunhyang University Seoul Hospital, Soonchunhyang University College of Medicine, Seoul, Republic of Korea; 5 Department of Nuclear Medicine, Konkuk University Medical Center, Konkuk University School of Medicine, Seoul, Republic of Korea; 6 Department of Thoracic and Cardiovascular Surgery, Samsung Medical Center, Sungkyunkwan University School of Medicine, Seoul, Republic of Korea; 7 Department of Radiology, Samsung Medical Center, Sungkyunkwan University School of Medicine, Seoul, Republic of Korea

**Keywords:** ^18^F-FDG, PET/CT, Oesophageal cancer, Lymph node metastasis, SUV_max_, CT attenuation

## Abstract

**Background:**

We sought to evaluate the diagnostic performance of fluorine-18-fluorodeoxyglucose positron-emission tomography/computed tomography (^18^F-FDG PET/CT) in the detection of metastatic lymph nodes by combined interpretation of PET/CT images in patients with oesophageal squamous cell carcinoma.

**Methods:**

Two hundred three patients with oesophageal squamous cell carcinoma underwent ^18^F-FDG PET/CT before oesophagectomy and lymph node dissection. Maximum standardized uptake value (SUV_max_), mean Hounsfield unit (HU), short axis diameter (size), and visual CT attenuation (high, iso-, low) were evaluated on noncontrast CT and PET images following PET/CT scan. In this combined interpretation protocol, the high attenuated lymph nodes were considered benign, even if the SUV_max_ value was high. The diagnostic accuracy of each method was compared using the postoperative histologic result as a reference standard.

**Results:**

A total of 1099 nodal stations were dissected and 949 nodal stations were proven to demonstrate metastasis. SUV_max_ and size of the malignant lymph nodes were higher than those of the benign nodes, and visual CT attenuation was significantly different among the two groups (*P* < 0.001). Using cutoff values of 2.6 for SUV_max_ and 10.2 mm for size, the combined interpretation of an SUV_max_ of more than 2.6 with iso- or low CT attenuation [area under the curve (AUC): 0.846, 95% confidence interval (CI): 0.824–0.867] showed significantly better diagnostic performance for detecting malignant lymph nodes than SUV_max_ only (AUC: 0.791, 95% CI: 0.766–0.815) and size (AUC: 0.693, 95% CI: 0.665–0.720) methods (*P* < 0.001) in a receiver operating characteristic curve analysis.

**Conclusions:**

The diagnostic accuracy of PET/CT for nodal metastasis in oesophageal squamous cell carcinoma was improved by the combined interpretation of ^18^F-FDG uptake and visual CT attenuation pattern.

## Background

Oesophageal cancer is the sixth most common cause of cancer-related mortality worldwide, leading to 3.2% of all deaths [1, 2]. The two most common histologic types found in more than 95% of all oesophageal cancer are squamous cell carcinoma (SCC) and adenocarcinoma. In particular, the SCC type has dominated for most of the twentieth century, particularly in Asian countries [3]. Surgical resection is the standard treatment approach for patients with early oesophageal cancer [4, 5]. Because the stage of oesophageal cancer is strongly associated with appropriate treatment options and its prognosis, accurate determination of clinical staging is essential. In particular, lymph node staging in oesophageal cancer is an important independent prognostic factor that can affect the surgical extent of lymph node dissection and the long-term survival of patients [6–10].

Lymph node metastasis in oesophageal cancer occur early and quickly to adjacent or regional lymph nodes because of the submucosal lymphatic drainage system of the oesophagus [11]. In cases of early-stage SCC, lymph node involvement is more widely distributed and the rates of metastatic lymph nodes are higher versus in those in adenocarcinoma [12, 13]. In a previous study, the prevalence of nodal metastasis differed significantly between SCC and adenocarcinoma cases (36% vs. 21%) in early oesophageal cancer [13]. Therefore, accurate lymph node staging is important in oesophageal carcinoma, especially in early-stage SCC.

For initial staging including the detection of nodal metastasis, fluorine-18-fluorodeoxyglucose (^18^F-FDG) positron emission tomography/computed tomography (PET/CT) has been widely used in patients with oesophageal cancer in recent years [14–17]. However, the results about the accuracy of regional nodal metastasis on ^18^F-FDG PET/CT are controversial, with relatively low sensitivity. Furthermore, in tuberculosis endemic areas, specificity for nodal staging has been reported to be reduced due to false-positive FDG-avid lymph nodes in oesophageal cancer [14, 18]. In several previous reports, mediastinal lymph nodes that show increased FDG uptake on PET images but high attenuation and/or calcification on the CT component of a PET/CT scan had a high probability for benign node in lung cancer [19, 20]. In other words, the combined interpretation of PET/CT images was helpful to improve the specificity for detecting metastatic lymph nodes in lung cancer. Recently, the usefulness of both measurements of X-ray attenuation taken from unenhanced CT images and FDG uptake in the PET/CT of lymph node metastasis in oesophageal cancer was reported [21]. However, this study was somewhat limited by its relatively small size and ambiguity of the PET criteria due to evaluation by visual analysis.

Therefore, the aim of this study was to evaluate the diagnostic performance of ^18^F-FDG PET/CT in detecting metastatic lymph nodes via the combined interpretation of PET/CT images in patients with oesophageal squamous cell carcinoma. Optimal PET/CT criteria for detecting metastatic lymph nodes were also investigated.

## Methods

### Patients

After this study was approved by the relevant Institutional Review Board (IRB no. 2019–03-129), the medical records of 479 consecutive patients with biopsy-proven oesophageal SCC who underwent ^18^F-FDG PET/CT for preoperative staging at our institute were retrospectively reviewed. Patients who underwent preoperative neoadjuvant chemotherapy or concurrent chemoradiation before surgery were excluded (*n* = 273). Also, three patients were excluded due to PET/CT data error. Finally, 203 patients who received oesophagectomy with lymph node dissection after PET/CT were finally included in this study. Recorded intervals between PET/CT scan and surgery ranged from one to 70 days (average 14 ± 10 days). Two patients had double primary cancer, specifically sigmoid colon and stomach cancer with a pathologic cell type of adenocarcinoma. Chest CT showed findings suggesting stable tuberculosis in 27 patients (13.3%).

### ^18^F-FDG PET/CT

Patients were fasted for at least 6 hours before the ^18^F-FDG PET/CT scan. Blood glucose levels before the injection of ^18^F-FDG were lower than 200 mg/dL in all patients. PET/CT imaging was performed using a dedicated PET/CT scanner (Discovery LS; GE Healthcare, Marlborough, MA, USA), without intravenous or oral contrast material.

Using the GE Discovery LS device (eight-slice helical CT scanner), a whole-body CT scan was obtained, using a continuous spiral technique (140 keV, 40–100 mA adjusted for the patient’s weight, section width of 5 mm), at 60 min after the injection of ^18^F-FDG (5.5 MBq/kg). After the CT scan, an emission scan from mid-thigh to the basal skull area was obtained for 4 minutes per frame in a two-dimensional mode. Attenuation-corrected PET images (voxel size: 4.3 × 4.3 × 3.9 mm) were reconstructed from the CT data, using an ordered-subset expectation maximization algorithm (28 subsets, two iterations). Attenuation-corrected torso PET/CT images were reviewed using the commercial software (GE Advantage Workstation; GE Healthcare, Marlborough, MA, USA).

### Image analysis

^18^F-FDG PET/CT images were retrospectively reviewed by two experienced nuclear medicine physicians, who were unaware of the clinical and pathological results, on a dedicated workstation by consensus. Lymph nodes were classified into 15-group nodal stations according to a modified lymph node mapping system for oesophageal cancer [22]. After determining the region of interest (ROI) of lymph nodes in the noncontrast CT image, the same size ROI was applied to the PET image. The physicians measured size (short axis diameter) and obtained the parameters of the mean Hounsfield unit (HU) and the maximum standardized uptake value (SUV_max_) of each lymph node using a ROI-based measurement on the noncontrast CT component and PET images from the PET/CT scan. By visual analysis of the noncontrast CT images of PET/CT, lymph nodes were classified into three groups, as follows: 1) lymph nodes with high attenuation, which had higher attenuation than that in the surrounding great vessels with (superior vena cava, ascending thoracic aorta and aortic arch) mediastinal window images or definite calcifications; 2) lymph nodes with iso-attenuation, which had similar attenuation to that that in the surrounding great vessels; and 3) lymph nodes with low attenuation, which had lower attenuation than that in the surrounding great vessels. In the combined interpretation of noncontrast CT component and FDG uptake on ^18^F-FDG PET/CT, benign lymph nodes were defined as those with high attenuation or calcification on noncontrast CT images, even if the SUV_max_ was higher than a cutoff value.

### Surgery and pathology

Transthoracic oesophagectomy and extensive lymph node dissection were performed by experienced thoracic surgeons. They dissected all visible and palpable lymph nodes in the surgical field after considering the results of preoperative imaging modalities including ^18^F-FDG PET/CT. Each dissected lymph node group was labelled according to a modified lymph node mapping system for oesophageal cancer [22]. Specimens were stained with haematoxylin and eosin and examined with optical light microscopy.

### Statistical analysis

Statistical analyses were performed using the Statistical Package for the Social Sciences version 22.0 (IBM Corp., Armonk, NY, USA) and the MedCalc version 14.8.1 (MedCalc Software, Mariakerke, Belgium). The histopathology of the surgical specimen was the gold standard to which the results of the imaging methods were compared and the accuracy of detecting lymph node involvement was calculated. To compare the PET and noncontrast CT parameters for benign and malignant lymph nodes, a Student’s t-test for continuous variables and the chi-squared test for categorical ones were used. Sensitivity, specificity, positive predictive values (PPVs), and negative predictive values (NPVs) were calculated for methods of PET/CT examinations on a lesion-per-lesion basis and were compared with outcomes of McNemar’s test. The predictive value of LN metastasis was measured via area under the curve (AUC) in a receiver operating characteristic (ROC) curve analysis, and the optimal cutoff values for detecting individual metastatic lymph nodes were determined. Differences were considered statistically significant when *P* values were less than 0.05.

## Results

### Lymph node histology

The patients’ characteristics are summarized in Table 1. A total of 1099 nodal stations were sampled in 203 patients (mean number of nodal stations sampled per patient: 5.4). Of these lymph nodes, 150 lymph nodes (13.6%) in 97 patients (47.8%) proved to be positive for malignancy. The metastasis sites were one of two lower cervical paratracheal (nodal station 1), 53 of 185 upper paratracheal (2), one of five prevascular and retrotracheal (3), none of 24 lower paratracheal (4), two of 100 aortopulmonary (5), 18 of 191 subcarinal (7), 18 of 38 thoracic paraesophageal (8), six of 130 pulmonary ligament (9), five of 68 tracheobronchial (10), two of 21 diaphragmatic (15), 16 of 72 paracardial (16), 17 of 112 left gastric (17), two of 65 common hepatic (18), one of one splenic (19), and eight of 85 celiac (20) nodes according to pathologic examination.

**Table 1:** Patient characteristics

Characteristics	Value
Age (years)	63 ± 8
Gender (male)	194 (95.6)
Locations	
Upper thoracic	14 (6.9)
Middle thoracic	58 (28.6)
Lower thoracic	118 (58.1)
Upper to mid-thoracic	1 (0.5)
Mid to lower thoracic	12 (5.9)
Histologic grade	
Well-differentiated	41 (20.2)
Moderate	108 (53.2)
Poor	21 (10.3)
Undetermined	33 (16.3)
Total	203

Data are presented as mean ± standard deviation or n (%)

### Comparisons of lymph node groups

When we examined the parameters of PET/CT scan in benign and malignant lymph node groups, the size and SUV_max_ of individual lymph nodes were significantly higher in the malignant lymph node group than in the benign group (*P* < 0.001). In the visual analysis of noncontrast CT attenuation, there were significant differences in low attenuation, iso-attenuation, and high attenuation and/or calcification between the benign and malignant lymph node groups (*P* < 0.001). The mean HU on noncontrast CT component was not statistically different between the groups (Table 2).

**Table 2:** Comparisons of lymph nodes between benign and malignant groups

	Benign (*n* = 949)	Malignant (*n* = 150)	*P* value
Size by CT (mm)	8.4 ± 3.0 (2.2–22.7)	11.1 ± 4.5 (4.4–36.87)	< 0.001
SUV_max_	2.2 ± 1.2 (0.6–8.9)	4.5 ± 3.0 (0.8–16.6)	< 0.001
CT attenuation pattern (visual)			< 0.001
Low attenuation	472	73	
Iso attenuation	308	74	
High attenuation and/or calcification	169	3	
CT attenuation	59.1 ± 66.0	58.2 ± 21.7	0.87

*CT* computed tomography, *SUV*_*max*_ Maximum standardized uptake value

Data are presented as mean ± standard deviation (min–max), number or mean ± standard deviation

### Diagnostic performance of individual node assessment for single modality

From the ROC analysis, the optimal cutoff points for SUV_max_ and size distinguishing benign from metastatic lymph nodes were 2.6 and 10.2 mm. The sensitivity and accuracy of an SUV_max_ of more than 2.6 for the detection of metastatic lymph nodes was significantly higher than those of size more than 10.2 mm, as follows: sensitivity of 72.7% (109/150) vs. 55.3% (83/150) and accuracy of 75.6% (831/1099) vs. 72.2% (793/1099), respectively (*P* ≤ 0.001). Although the specificity of SUV_max_-only assessment was higher than that of size measurement (76.1% [722/949] vs. 74.8% [710/949]), the difference was not statistically significant (*P* = 0.476) (Table 3).

**Table 3:** Diagnostic performance for detecting regional lymph node metastasis

	Sensitivity (%)	Specificity (%)	PPV (%)	NPV (%)	Accuracy (%)
SUV_max_ > 2.6^a^	72.7 (65.4–79.1)^b^	76.1 (74.9–77.1)	32.4 (29.2–35.3)	94.6 (93.2–95.9)	75.6 (73.6–77.4)
Size > 10.2 mm^a^	55.3 (47.7–62.7)	74.8 (73.6–76.0)	25.8 (22.2–29.2)	91.4 (89.9–92.8)	72.2 (70.1–74.2)
CT attenuation pattern (visual)	98.0 (94.0–99.5)	17.8 (17.2–18.0)	15.9 (15.2–16.1)	98.3 (94.7–99.5)	28.8 (27.7–29.2)

*CT* computed tomography, *NPV* negative predictive value, *PPV* positive predictive value, *SUV*_*max*_ Maximum standardized uptake value

^a^Data were evaluated using cutoff value from ROC analysis

^b^Numbers in parentheses are 95% confidence intervals

Sensitivity: SUV_max_ vs. size, *P* = 0.001; SUV_max_ vs. CT attenuation pattern, *P* < 0.001; size vs. CT attenuation pattern, *P* < 0.001

Specificity: SUV_max_ vs. size, *P* = 0.476; SUV_max_ vs. CT attenuation pattern, *P* < 0.001; size vs. CT attenuation pattern, *P* < 0.001

Accuracy: SUV_max_ vs. size, *P* < 0.001; SUV_max_ vs. CT attenuation pattern, *P* < 0.001; size vs. CT attenuation pattern, *P* < 0.001

By visual analysis on noncontrast CT component, the sensitivity, specificity, and accuracy of attenuation patterns were 98.0% (147/150), 17.8% (159/949), and 28.8% (793/1099). In the visual CT attenuation analysis, the sensitivity was significantly higher than the methods of SUV_max_ and size evaluation (*P* < 0.001), while the specificity and accuracy were significantly lower than those methods (*P* < 0.001) (Table 3).

With ROC curve analysis, the diagnostic performance of SUV_max_ with a cutoff value of 2.6 [AUC: 0.791, 95% confidence interval (CI): 0.766–0.815] for distinguishing benign from metastatic nodes was significantly better than that of size with a cutoff value of 10.2 mm (AUC: 0.693, 95% CI: 0.665–0.720), respectively (*P* < 0.001).

### Combined interpretation of PET/CT images

**Fig. 1:**
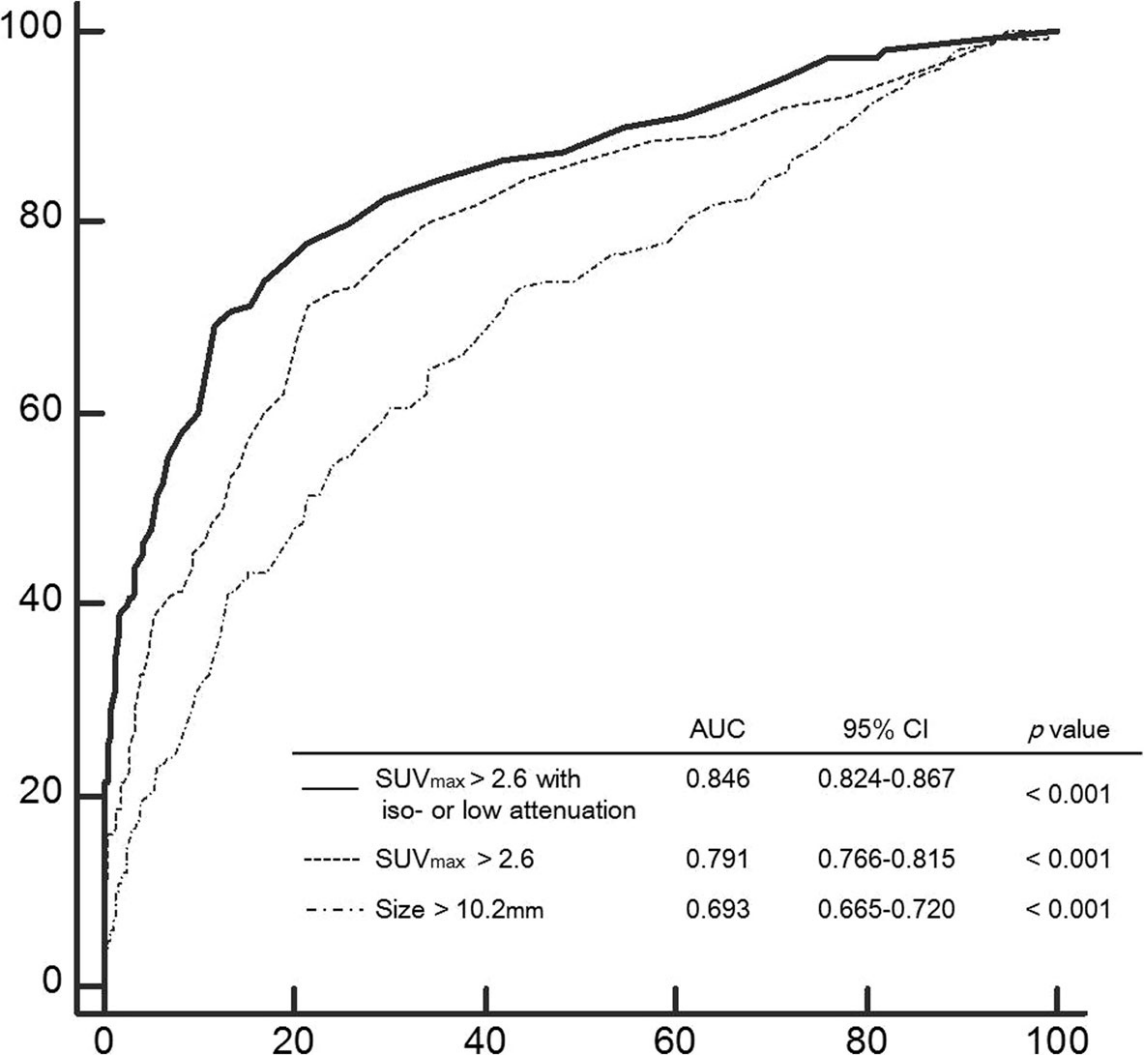
ROC curve for combined interpretation, SUV_max_, and size for detecting metastatic lymph nodes in oesophageal SCC. *ROC* receiver operating characteristic, *SCC* squamous cell carcinoma, *SUV*_*max*_ Maximum standardized uptake value

For the combined interpretation of PET/CT images, 172 lymph nodes with visually high attenuation were converted to benign. The greatest test accuracy was obtained by selecting a cutoff value of SUV_max_ of 2.6 (AUC: 0.846, 95% CI: 0.824–0.867; *P* < 0.001) with a sensitivity of 70.7% (104/150), specificity of 86.7% (823/949), and accuracy of 84.5% (929/1099), respectively (Table 4). In the combined interpretation of PET/CT images, the sensitivity was decreased as compared with in SUV_max_-only evaluation, but there was no significant difference between the two methods (*P* = 0.25). Additionally, the specificity and accuracy were significantly improved in the combined interpretation method versus in the single assessment methods (*P* < 0.001). A comparison of the obtained ROC curves showed a significant statistical difference in AUC among the combined interpretation of SUV_max_ of more than 2.6 with iso- and low attenuation and SUV_max_ of more than 2.6 only and size measurement (*P* < 0.001) (Fig. 1).

**Table 4:** Diagnostic performance of combined interpretation of PET/CT images

	Sensitivity (%)	Specificity (%)	PPV (%)	NPV (%)	Accuracy (%)
SUV_max_ > 2.6^a^ with iso- or low CT attenuation	70.7 (63.6–77.0)^b^	86.7 (85.6–87.7)	45.7 (41.1–49.8)	94.9 (93.7–96.0)	84.5 (82.6–86.3)

*CT* computed tomography, *NPV* negative predictive value, *PET-CT* positron-emission tomography/computed tomography, *PPV* positive predictive value, *SUV*_*max*_ Maximum standardized uptake value

^a^Data were evaluated using cutoff value from ROC analysis

^b^Numbers in parentheses are 95% confidence intervals

## Discussion

**Fig. 2:**
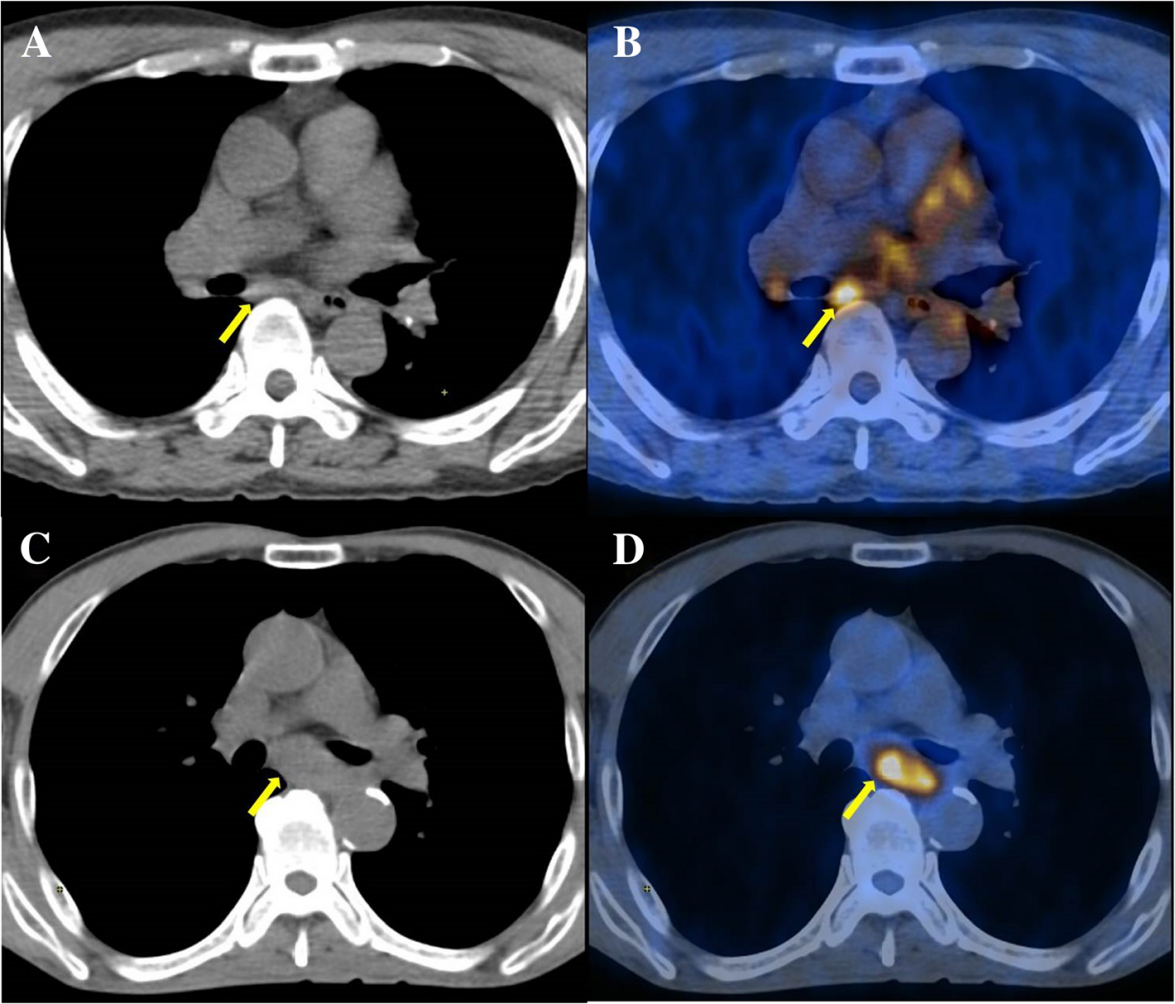
PET/CT image of a 64-year-old male with oesophageal cancer showed lymph node enlargement in subcarinal area. The lymph node’s CT attenuation was high as 112 HU (**a**) with SUV_max_ of 4.4 (**b**), and this lesion was revealed to be benign lymph node after surgery. Meanwhile, PET/CT image of a 65-year-old male oesophageal cancer patient with lymph node enlargement in subcarinal area showed a low CT attenuation of 23 HU (**c**) with a SUV_max_ of 4.4 (**d**) which revealed to be a metastatic lymph node. *CT* computed tomography, *SUV*_*max*_ Maximum standardized uptake value

We performed a study to determine the diagnostic performance of ^18^F-FDG PET/CT in the detection of individual lymph node metastasis in patients with oesophageal SCC. The combined interpretation of SUV_max_ on PET images with CT attenuation on noncontrast CT component was more accurate than that single assessment of SUV_max_, size, or CT attenuation of PET/CT images. When lymph node status determined by SUV_max_ was more than 2.6 with iso-attenuation or low attenuation on noncontrast CT images, the accuracy was improved in detecting individual lymph node metastasis in patients with oesophageal SCC (Fig. 2). The specificity was also increased in the combined interpretation of PET/CT images compared to the single evaluated methods, although sensitivity was decreased.

Lymph node involvement in oesophageal cancer has a great impact on both treatment decision and prognosis of the patient. To detect such lymph node metastasis of the patient diagnosed by oesophageal cancer, pretreatment evaluations have used many modalities including endoscopic ultrasonography (EUS), chest CT, and ^18^F-FDG PET/CT. Meta-analysis studies have revealed that EUS is the most sensitive of these options, whereas CT and FDG PET are more specific tests for the detection of regional lymph node metastasis but overall offer similar diagnostic performance [23, 24]. EUS has limitations for evaluating the nodal status of oesophageal cancer patients with the presence of stenosis or strictures, which may affect low accuracy and nodal overstaging. In addition, interobserver variation and machine-dependent factors may exist and patient cooperation is essential for safe and successful EUS practice [25].

Chest CT is the most commonly used imaging technique for the staging of oesophageal cancer and helps to evaluate lymph nodes dependent on size criteria. Because the criteria of enlarged lymph nodes vary according to anatomical location and the detection of metastasis in normal-sized lymph nodes, the sensitivity and specificity of nodal metastasis assessment are low and remain a challenge [24]. In our study, the size on ^18^FDG PET/CT was significantly different between the benign and malignant groups, but the scores of sensitivity and accuracy were poor at 55.3 and 69.3% and the diagnostic performance was significantly lower than that of either SUV_max_ only or combined interpretations. This is supported by several reports that benign nodes might be enlarged as a result of benign processes such as reactive hyperplasia, obstructive pneumonia, or atelectasis, whereas metastatic nodes may appear normal in size if metastasis is present [26, 27].

Previous studies have evaluated the accuracy of oesophageal cancer lymph node staging by using PET/CT. Hsu, et al. reported that although positive nodal FDG uptake on PET/CT was related to the pathologic findings of regional lymph node, the ability of PET/CT to predict lymph node metastasis remained inadequate [28]. Jeong, et al. also found PET/CT missed nodal metastasis detection in more than half of the patients with early stage oesophageal SCC [29]. A recent meta-analysis study indicated the ^18^F-FDG PET/CT for detecting regional lymph node metastasis had a sensitivity and specificity with 95% CI of 62% (40–79) and 96% (93–98) [30]. In other meta-analysis, the pooled sensitivity and specificity for PET/CT were 0.59 (0.53–0.64) and 0.81 (0.74–0.86) [24]. Therefore, lymph node staging on PET/CT is challenging because it has some limitations in the detection of regional lymph node metastasis. Our study showed a similar performance with a sensitivity and specificity of 72.7 and 76.1%, retrospectively, and the ability of SUV assessment to predict node metastasis was deemed unsatisfactory. When microscopic lymph node metastasis is present, the ^18^F-FDG uptake of the lymph node may not be increased on PET/CT, whereas inflammatory lymph nodes are shown to be falsely increased in various FDG uptakes on PET/CT, making it difficult to distinguish such from malignancy. Budiawan, et al. found that the mean SUV_max_ of inflammatory/benign lymph nodes in lung cancer patient was 4.96 ± 2.08 (range 2.20–11.22) [31]. In Lin, et al. study, the SUV_max_ of pathologically negative lymph nodes in lung cancer was 7.34 ± 6.1 [32]. These nodes showed follicular hyperplasia in the cortex and anthracitic pigmentation and macrophage infiltration in the medulla, and these inflammatory changes can provoke high false-positive rates and low specificity with ^18^F-FDG PET/CT [18, 20]. Although 27 patients with pulmonary tuberculosis history on CT images were enrolled in the present study, these patients did not affect the results of the study.

To check the best performance of ^18^F-FDG PET/CT for detecting individual nodal involvement of metastasis, we included the data from the without contrast enhancement and did not include chest CT data. The noncontrast CT component of PET/CT images, which yields X-ray attenuation values, can be helpful for characterizing lymph nodes because higher attenuations than those of mediastinal structures with/without containing calcification may be observed in inflammatory lymph nodes [20, 33]. Therefore, several reports have suggested that lymph nodes with high attenuation and/or containing calcification on noncontrast CT images, even if they show increased FDG uptake on PET, should be regarded as benign, especially where chronic granulomatous disease is endemic [19, 20]. However, our study revealed there was no significant difference between benign and malignant lymph nodes in terms of mean HU, while conversely the visual attenuation pattern was significantly different between them. This result is discordant with those of a previous study that revealed a significant difference for mean HU between the malignant and benign lymph nodes groups [21]. This might be due to their small number of study subjects. This result is also the reason for why we considered visually high-attenuated lymph nodes as benign, unlike in previous studies which usually employed a mediastinal structure to 70 HU criterion for the high attenuation value.

In the present study, when interpreting ^18^F-FDG PET/CT, combined interpretation considering SUV_max_ on PET images with visual attenuation assessment on noncontrast CT component was significantly more specific than SUV_max_-only evaluation on PET images for the detection of regional lymph node metastasis without a significant difference in sensitivity. The overall diagnostic performance for the SUV_max_-only method in our and previous studies was around 70% (range: 63–73.1%), which significantly increased to 84.5% in the combined interpretation of an SUV_max_ of more than 2.6 with iso- or low CT attenuation. Kim et al., who had similar study settings, showed concordance with our study, but they couldn’t obtain the optimal SUV_max_ because of PET image evaluation by visual analysis (12). They also concluded that the assignment of highly attenuated lymph nodes with increased FDG uptake as benign could improve diagnostic accuracy for metastatic lymph nodes. Therefore, performing the combined interpretation considering FDG uptake and attenuation on ^18^F-FDG PET/CT could improve the specificity of lymph node staging of oesophageal cancer by reducing false-positives and could influence the surgical extent of lymphadenectomy, especially in populations where chronic granulomatous disease is endemic.

Our study possesses several limitations. First, it is a retrospective study and we included only patients who had underwent oesophagectomy with lymph node dissection. Thus, we might have selection bias and both the sensitivity and accuracy may have been underestimated. Second, because oesophageal adenocarcinoma is very rare in Asia including our country, this study included patients with SCC only. Therefore, further study is needed for patients with oesophageal adenocarcinoma to investigate whether our results can be applied for those patients. Third, this study was localized to a single hospital; therefore, further multicentre and prospective studies may be needed to evaluate the value of PET/CT for regional nodal metastasis in oesophageal cancer patients. Finally, because the optimal cutoff values of mean SUV_max_ and size for individual lymph nodes were calculated by the sum of maximum sensitivity and specificity, the performance ability may be overestimated.

## Conclusions

The diagnostic accuracy of PET/CT for nodal metastasis in oesophageal squamous cell carcinoma is improved by the combined interpretation of ^18^F-FDG uptake and visual CT attenuation pattern. In other words, when ^18^F-FDG-avid lymph nodes of more than a SUV_max_ of 2.6 with iso- or low CT attenuation were considered as metastasis, and nodes that showed high CT attenuation and/or calcification were regarded as benign even if SUV_max_ was 2.6 or higher, the accuracy is enhanced by reducing the false-positive rate in endemic areas of granulomatous disease. The high specificity of the combined interpretation of SUV_max_ and visual CT attenuation on PET/CT images for nodal assessment may be helpful in determining therapeutic plans in patients with oesophageal SCC.

## Data Availability

The datasets used and/or analysed during the current study are available from the corresponding author on reasonable request.

## Abbreviations

^18^F-FDG: fluorine-18-fluorodeoxyglucose
AUC: area under the curve
CI: confidence interval
EUS: endoscopic ultrasonography
HU: Hounsfield unit
MBP: mediastinal blood pool
NPV: negative predictive values
PET/CT: positron emission tomography/computed tomography
PPV: positive predictive value
ROC: receiver operating characteristic
ROI: region of interest
SCC: squamous cell carcinoma
SUV_max_: maximum standardized uptake value
